# Renin–Angiotensin System Autoantibody Network in Parkinson’s Disease Patients

**DOI:** 10.3390/antiox14060706

**Published:** 2025-06-10

**Authors:** Carmen M. Labandeira, Laura Camacho-Meño, Paula Aracil-Pastor, Juan A. Suárez-Quintanilla, Jose L. Labandeira-García, Ana I. Rodríguez-Pérez

**Affiliations:** 1 Neurology Service, University Hospital of Ourense, 32005 Ourense, Spain; carmen.maria.labandeira.guerra@sergas.es; 2 Cellular and Molecular Neurobiology of Parkinson’s Disease, Research Center for Molecular Medicine and Chronic Diseases (CIMUS), University of Santiago de Compostela, 15782 Santiago de Compostela, Spain; laura.camacho.meno@usc.es (L.C.-M.); paula.aracil.pastor@usc.es (P.A.-P.); 3 Unidad de Atención Primaria, Centro de Saúde Fontiñas, University of Santiago de Compostela, 15782 Santiago de Compostela, Spain; juanantonisuarez.suarez@usc.es; 4 Instituto de Investigación Sanitaria de Santiago de Compostela (IDIS), 15706 Santiago de Compostela, Spain; 5 Networking Research Center on Neurodegenerative Diseases (CIBERNED), 28029 Madrid, Spain

**Keywords:** aging-related changes, angiotensin receptors, autoimmunity, biomarkers, cytokines, G protein-coupled receptors, neuroinflammation, oxidative stress, Parkinson’s disease, sex differences

## Abstract

The tissue renin–angiotensin system (RAS) is a regulator of oxidative and inflammatory homeostasis by balancing its pro-oxidative/pro-inflammatory axis (angiotensin II, AngII, and AngII type-1 receptor, AT1) and its anti-oxidative/anti-inflammatory axis (AngII/AT2 and ACE2/Ang1-7/Mas receptors). An RAS dysregulation contributes to diseases, including Parkinson’s disease (PD). Immune mechanisms are involved in PD. An increase in levels of pro-oxidative/pro-inflammatory autoantibodies for AT1 (AT1-AAs) and ACE2 (ACE2-AAs) has been recently observed in PD. However, it is not known whether dysregulation of autoantibodies for AT2, MasR, and the correlations among different RAS-AAs occurs in PD. In 106 controls and 117 PD patients, we used enzyme-linked immunosorbent assays to determine correlations among serum RAS-AAs, and among RAS-AAs and pro-inflammatory cytokines and 27-hydroxycholesterol. PD patients showed an increase in MasR-AAs, and a more interconnected cluster of correlations among RAS-AAs (AT1-AA, AT2-AA, MasR-AA, ACE2-AA), changes in RAS-AA networks with sex and age, and differences in networks between RAS-AAs and major PD-related pro-inflammatory cytokines and 27-hydroxycholesterol. The association between AT1-AAs and PD remained significant even after adjustment for age and other variables. This study reveals a disease-specific network of RAS autoantibodies in PD that links immune and oxidative pathways and identifies new biomarker patterns and potential therapeutic targets.

## 1. Introduction

The tissue or local renin–angiotensin system (RAS) is a regulator of oxidative and inflammatory homeostasis in different tissues and organs, including the brain, by balancing two RAS opposite arms [1,2]: the pro-oxidative/pro-inflammatory axis, mediated by angiotensin II (Ang II) acting on angiotensin type 1 (AT1) receptors, and the anti-inflammatory/anti-oxidative axis, mediated by the activation of angiotensin type 2 (AT2) receptors and Mas receptors (MasR). Angiotensin-converting enzyme 2 (ACE2) transforms components of the pro-inflammatory axis (particularly, AngII) into components of the compensatory axis such as angiotensin-(1-7) (Ang1-7) that binds to the MasR [3,4]. An RAS dysregulation towards the pro-oxidative/pro-inflammatory axis contributes to the progression of many peripheral and brain diseases, including neurodegenerative diseases such as Parkinson’s disease (PD) [2,5]. In humans, recent studies revealed that high AT1 receptor gene (*AGTR1*) expression identifies the most vulnerable human dopaminergic neurons [6,7,8] and that treatment with AT1 blockers decreases the risk of PD development [9,10,11].

Growing evidence supports a key role for immune mechanisms in PD pathophysiology [12]. In PD models and PD patients, we have recently shown an increase in serum levels of autoantibodies for AT1 receptors (AT1-AAs) [13,14]. AT1-AAs have a strong agonistic effect on AT1 receptors [15,16,17,18] and enhance dopaminergic neurodegeneration through several mechanisms, such as promoting oxidative stress and neuroinflammation, intraneuronal calcium raising, alpha–synuclein aggregation, and blood–brain barrier disruption, which were mitigated by treatment with AT1 receptor blockers [13,14,19]. In PD models and PD patients, we also observed increased levels of ACE2 autoantibodies (ACE2-AAs) [13,14]. ACE2-AAs impair ACE2 activity [20,21], consequently reducing the production of Ang1-7, and weakening the protective signaling of the anti-oxidative/anti-inflammatory AT2 and Mas receptors [22]. However, it is not known whether the dysregulation of the agonistic [22,23] AT2 and Mas receptor autoantibodies (AT2-AAs, MasR-AAs) and the correlations among different RAS autoantibodies occur in PD patients as compared with non-PD controls.

The presence of endogenous self-reactive autoantibodies that recognize a large variety of G-protein coupled receptors (GPCRs), including the above-mentioned RAS receptors, is well established. Their increase has been associated with several cardiovascular, neurological, and autoimmune diseases [24], as most AAs are functional and activate GPCR signaling, uncoupling receptors from the endogenous signaling networks [24]. However, GPCR-AAs are also present in serum from healthy subjects, although in lower concentrations than in patients with autoimmune-mediated disorders [25,26]. It has been suggested that autoantibodies targeting GPCRs are involved in immune system homeostasis and neuroimmune communication [25,26], and the term antibodiom has been proposed [27]. However, autoantibody correlations may break down, leading to changes in signatures of AA concentrations compared with those of healthy controls, promoting autoimmune diseases. Conversely, diseases, inflammation, and tissue injuries may also alter autoantibody correlation, and sex and age may also modulate correlations [25,26].

In the work reported here, we investigated whether levels of AAs for receptors of the RAS anti-oxidative/anti-inflammatory axis are dysregulated in PD patients in comparison with non-PD controls. We also studied the possible changes in correlation signatures of RAS AAs in PD patients relative to controls, possible effects of sex and age, and possible predictive value of these components as markers of PD status. Finally, we analyzed possible correlations of PD and control RAS AA networks with major pro-inflammatory cytokines such as interleukin-17 (IL-17), interleukin-6 (IL-6), tumor necrosis factor superfamily member 14 (TNFSF14, LIGHT), tumor necrosis factor-alpha (TNF-α), and the pro-oxidative lipid marker 27-hydroxycholesterol (27-OHC), previously associated with increased risk of PD [28,29,30,31]. Our hypothesis suggests the presence of a disease-specific network of RAS autoantibodies that may correlate with markers of neuroinflammation and oxidative stress, revealing new biomarker patterns and potential therapeutic targets in PD.

## 2. Materials and Methods

### 2.1. Description of the Study Population

The present analysis is based on a sample previously collected [13] and subsequently expanded with additional measurements. The study population included 117 adult patients diagnosed with PD and 106 adult control subjects. Control participants were recruited from among individuals attending a dental clinic at the Primary Health-Care Unit Fontiñas (Santiago de Compostela, Spain). PD patients were enrolled between June 2018 and November 2019 at the Álvaro Cunqueiro University Hospital Complex (Vigo, Spain). The study was approved by the Galician Drug Research Ethics Committee (CEIm-G; protocol 2017/590) and conducted in accordance with the principles outlined in the Declaration of Helsinki. Detailed demographic and clinical characteristics of both PD patients and control subjects at the time of assessment are provided in [13]. Inclusion criteria for PD patients were a confirmed diagnosis of PD according to the clinical diagnostic criteria of the UK Parkinson’s Disease Society Brain Bank [32], age over 40 years, voluntary participation, and provision of written informed consent. Control subjects were required to have no diagnosis of PD or other neurodegenerative disorders and to participate voluntarily after providing written informed consent. Initially, 121 PD patients and 114 controls were recruited. However, individuals with comorbidities known to influence AT1-AA levels (e.g., liver transplantation, APOE4 carrier status, multiple sclerosis) or with incomplete medical records were excluded. The final study sample comprised 117 PD patients and 106 control subjects.

### 2.2. Sample Collection and Storage

At the time of recruitment, peripheral blood was collected by venipuncture into Vacutainer^®^ SST™ II Advance serum separator tubes (8.5 mL, Ref. 366468; Becton Dickinson, Franklin Lakes, NJ, USA). Samples were centrifuged at 1500× *g* for 20 min at room temperature to isolate serum. The resulting supernatant was aliquoted into cryovials and stored at −80 °C until further analysis. Serum samples were subsequently used for the quantification of autoantibodies against the angiotensin II type 1 receptor (AT1-AA), angiotensin-converting enzyme 2 (ACE2-AA), angiotensin II type 2 receptor (AT2-AA), and the Mas receptor (MasR), as well as for the measurement of interleukin-17 (IL-17), interleukin-6 (IL-6), tumor necrosis factor superfamily member 14 (TNFSF14, also known as LIGHT), tumor necrosis factor-alpha (TNF-α), and 27-hydroxycholesterol (27-OHC).

### 2.3. Clinical Follow-Up and Data Collection

All participants were longitudinally followed through an institutional electronic health information system until July 2021. Clinical data were extracted retrospectively from electronic medical records and included demographic characteristics, comorbidities, presenting symptoms, physical examination findings, radiological results, disease stage, administered treatments, and laboratory test results.

### 2.4. Quantification of Autoantibodies

Serum concentrations of AT1-AA, ACE2-AA, AT2-AA, and MasR autoantibodies were determined using commercially available solid-phase sandwich ELISAs (catalog numbers 12,000, 16,000, 16,200 and 17,000, respectively; CellTrend, Luckenwalde, Germany), following the manufacturer’s protocols. Absorbance was measured at 450/620 nm using a multi-well plate reader (Infinite M200, TECAN; software version 2.0). Autoantibody concentrations were interpolated from standard curves generated by a four-parameter logistic (4PL) regression model. Samples with absorbance values exceeding the upper limit of the standard curve were appropriately diluted with assay buffer and reanalyzed to obtain values within the dynamic range of the assay.

### 2.5. Quantification of Cytokines and 27-Hydroxycholesterol

Serum concentrations of IL-17, IL-6, TNFSF14 (also known as LIGHT), TNF-α, and 27-OHC were quantified using human-specific ELISA kits, in strict accordance with the manufacturer’s instructions. The following kits were employed: IL-17 (Cat# BMS2017HS), IL-6 (Cat# BMS213HS), LIGHT (Cat# BMS2218), and TNF-α (Cat# BMS223HS) from Invitrogen (Thermo Fisher Scientific, Waltham, MA, USA), and 27-OHC (Cat# LS-F40084) from LSBio (LifeSpan BioSciences, Seattle, WA, USA). All assays were performed on serum samples in duplicate. Absorbance was measured at 450 nm with wavelength correction at 620 nm using a microplate spectrophotometer (Infinite M200, TECAN, Tecan Group Ltd., Männedorf, Switzerland), and analyte concentrations were calculated by interpolation from standard curves fitted with a 4PL regression model.

### 2.6. Statistical Analysis

Sample size estimations were conducted *a priori* for each primary study objective, assuming a significance level (α) of 0.05 and a statistical power (1 − β) of 0.80. For the correlation analysis, a minimum of 29 participants was required to detect a large effect size (Pearson’s *r* = 0.50) between biomarker concentrations. According to Cohen’s criteria (1988), an absolute value of *r* = 0.10 is considered small, *r* = 0.30 medium, and *r* = 0.50 large [33]. For the group comparison, an estimated effect size (d = 0.419) was derived from previously published data [14] on differences in AT1-AA levels between PD patients and healthy controls, resulting in a required sample size of 104 participants per group. To account for potential deviations from normality, a correction factor of 15% was applied to the final sample size estimate.

Descriptive statistics are presented as medians and interquartile ranges (IQR) for continuous variables, and frequencies and percentages for categorical variables. The assumption of normality was tested using the Anderson–Darling test, and homogeneity of variances was assessed with the Fligner–Killeen test. For two-group comparisons, Student’s *t*-test was applied when both normality and homoscedasticity assumptions were satisfied; in cases of unequal variances, Welch’s *t*-test was employed; and for non-normally distributed variables, the Mann–Whitney U test was used. Categorical variables were compared using the Pearson chi-square test. Bivariate associations between continuous biomarkers were evaluated using Pearson’s correlation coefficient. Correlation matrices were visualized using correlograms and Sankey (alluvial) plots. To explore underlying structure in the biomarker correlations, unsupervised hierarchical clustering was performed using complete linkage and Euclidean distance metrics. To assess the multivariate association between biomarker profiles, clinical covariates, and PD diagnosis, binary logistic regression models were fitted, with group assignment (PD vs. control) as the dependent variable. Covariates yielding a *p*-value < 0.10 in univariate analyses were considered for inclusion in the multivariable model. Stepwise variable selection based on Akaike Information Criterion (AIC) was subsequently applied. The dataset was randomly partitioned into a training set (80%) for model fitting and a test set (20%) for performance evaluation. Predictive accuracy was assessed using the area under the receiver operating characteristic curve (AUC), sensitivity, specificity, and overall classification accuracy. Model calibration was evaluated using the Hosmer–Lemeshow goodness-of-fit test. All statistical analyses were performed using R version 4.4.2 [34]. A two-tailed *p*-value < 0.05 was considered statistically significant.

## 3. Results

### 3.1. Patient Characteristics

The study included 106 neurologically and cognitively healthy control participants (mean age  =  64.9  ±  9.25 Standard Deviation [SD]; 51 men, 55 women) and 117 patients with a confirmed clinical diagnosis of PD (mean age  =  69.43  ±  10.09 SD years; 57 men, 60 women). A detailed description of the demographic and clinical characteristics of both PD and control groups, as well as comprehensive information on the inclusion criteria and sample collection procedures, is provided in the Methods section and further detailed in our previous publication [13].

### 3.2. Autoantibodies for Components of Renin–Angiotensin System in Controls and Parkinson’s Disease Patients

**Figure 1:**
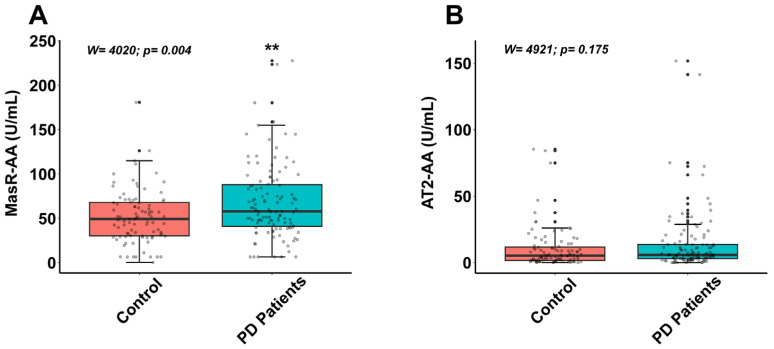
Serum levels of autoantibodies targeting components of the renin–angiotensin system in control subjects and Parkinson’s disease (PD) patients. (**A**) Mas receptor-activating autoantibodies (MasR-AAs) were significantly elevated in PD patients compared to controls (*p* = 0.004; Wilcoxon–Mann–Whitney test). (**B**) No significant differences were observed in the levels of angiotensin II type 2 receptor-activating autoantibodies (AT2-AA) between groups (*p*  =  0.175; Wilcoxon–Mann–Whitney test). Data distribution is shown using a box plot, where boxes represent the interquartile range (IQR) and the median (black line), and whiskers represent ± 1.5 IQR. Each point represents an individual subject. ** *p*  <  0.01.

Our previous findings demonstrated that PD patients exhibited significantly higher levels of AT1-AAs and ACE2-AAs compared to control individuals (see [13] and Supplementary Figure S1). However, potential differences in autoantibody levels for AT2 and Mas receptors, as well as their associated regulatory networks, had not been previously investigated. In the present study, we addressed these gaps and found that median serum concentrations of MasR-AAs were 49.11 U/mL [interquartile range (IQR): 29.98–67.69] in the control group and 57.67 U/mL [IQR: 40.66–87.77] in PD patients. For AT2-AAs, median values were 5.32 [IQR 1.59–11.73] U/mL in controls and 5.83 [IQR 3.02–13.65] U/mL in the PD group. Wilcoxon–Mann–Whitney test analysis revealed significantly higher serum levels of MasR-AAs in PD patients compared to controls (*p*  <  0.01; W  =  4020), while no significant difference was observed for AT2-AAs (*p* > 0.05; W  =  4921) (Figure 1).

### 3.3. Correlation Networks of RAS Autoantibodies in Controls and Parkinson’s Disease Patients

**Figure 2:**
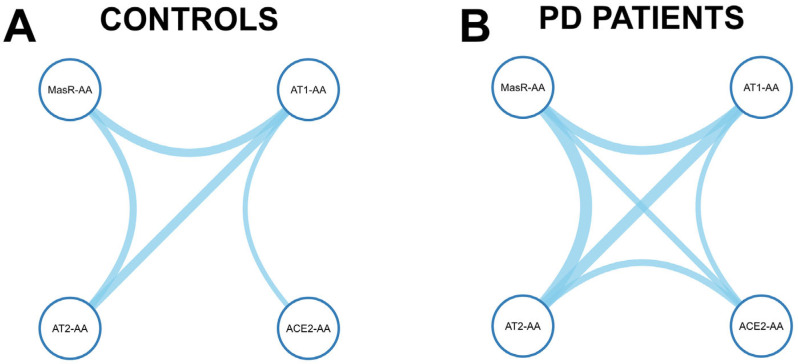
Spearman correlation networks of autoantibodies targeting renin–angiotensin system (RAS) components in healthy controls (**A**) and Parkinson’s disease (PD) patients (**B**). Nodes represent autoantibodies for AT1, AT2, Mas receptors, and ACE2. Edges indicate significant correlations (*p* < 0.05), with line thickness proportional to the correlation strength (r_s_).

Pairwise Spearman correlation analysis among RAS-related autoantibodies revealed a denser and more integrated network in PD patients compared to healthy controls. In controls (Supplementary Table S1; Figure 2A), AT1-AAs showed moderate correlations with AT2-AAs (r_s_ = 0.367, 95% CI: 0.173–0.533, *p* < 0.001) and MasR-AAs (r_s_ = 0.341, 95% CI: 0.139–0.515, *p* = 0.001), while its correlation with ACE2-AAs was weaker and marginally significant (r_s_ = 0.204, 95% CI: −0.002–0.394, *p* = 0.046). Additionally, a significant correlation was observed between AT2-AAs and MasR-AAs (r_s_ = 0.288, *p* = 0.006). No significant associations were detected between ACE2-AAs and the other RAS components. In contrast, PD patients (Supplementary Table S2; Figure 2B) exhibited a more interconnected cluster among the four RAS-AAs (AT1-AAs, AT2-AAs, MasR-AAs, ACE2-AAs), characterized by stronger and statistically significant correlations across all pairwise comparisons. AT1-AAs were significantly associated with AT2-AAs (r_s_ = 0.460, *p* < 0.001), MasR-AAs (r_s_ = 0.355, *p* < 0.001), and ACE2-AAs (r_s_ = 0.215, *p* = 0.020). Additionally, a significant correlation was observed between AT2-AAs and MasR-AAs (r_s_ = 0.491, *p* < 0.001). Notably, correlations involving ACE2-AAs became significant only in the patient group, with positive associations with AT2-AAs (r_s_ = 0.274, *p* = 0.003) and MasR-AAs (r_s_ = 0.257, *p* = 0.006). These findings reflect enhanced coordination among RAS autoantibody levels in PD.

### 3.4. Sex-Dependent Correlation Networks of RAS Autoantibodies in Controls and Parkinson’s Disease Patients

**Figure 3:**
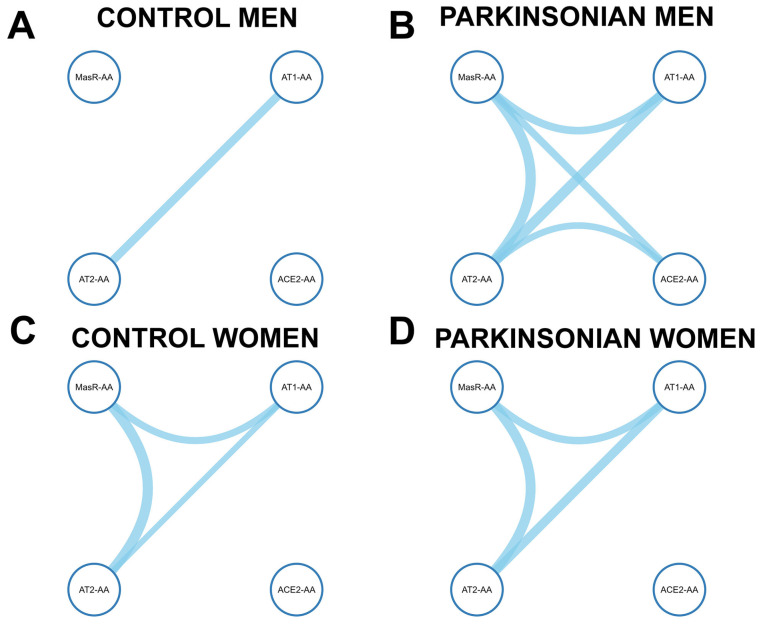
Sex-stratified Spearman correlation networks of RAS-related autoantibodies in Parkinson’s disease (PD) patients and controls. Analyses were performed separately in men and women. Nodes represent autoantibodies for AT1, AT2, Mas receptors, and ACE2. Edges show significant correlations (*p* < 0.05), with line thickness indicating strength (r_s_).

Given evidence of sex-related differences in immune regulation, we next explored whether the association patterns among RAS-related autoantibodies varied by sex (Figure 3). We performed stratified correlation analyses in men and women, separately for PD patients and controls, to identify potential sex-specific network differences that could contribute to the biological heterogeneity of PD.

Sex-stratified Spearman correlation analysis revealed distinct RAS autoantibody interaction patterns both in controls and PD patients. Among control men (Figure 3A; Supplementary Table S3), only a single significant correlation was detected between AT1-AAs and AT2-AAs (r_s_ = 0.396, *p* = 0.006), with all other pairwise interactions falling below significance thresholds. Notably, ACE2-AAs remained functionally uncoupled in this subgroup. In contrast, in control women (Figure 3C; Supplementary Table S4), AT1-AAs displayed statistically significant correlations with AT2-AAs (r_s_ = 0.280, *p* = 0.049) and with MasR-AAs (r_s_ = 0.337, *p* = 0.02). A more robust correlation was observed between AT2-AAs and MasR-AAs (r_s_ = 0.488, *p* = 0.001). The correlation between AT1-AAs and ACE2-AAs was weaker and not statistically significant (r_s_ = 0.183, 95% CI: −0.082–0.424, *p* = 0.161), and no significant associations were found between ACE2-AAs and the remaining RAS components. Parkinsonian men (Figure 3B; Supplementary Table S5) presented a markedly integrated RAS autoantibody network. Strong correlations were observed between AT1-AAs and AT2-AAs (r_s_= 0.494, *p* < 0.001), AT1-AAs and MasR-AAs (r_s_ = 0.370, *p* = 0.005), ACE2-AAs and AT2-AAs (r_s_ = 0.317, *p* = 0.017), and ACE2-AAs and MasR-AAs (r_s_ = 0.352, *p* = 0.008). Additionally, a significant correlation was observed between AT2-AAs and MasR-AAs (r_s_ = 0.492, *p* < 0.001). This pattern reflects a coordinated upregulation of RAS elements, indicative of an altered regulatory state associated with the man PD phenotype. In parkinsonian women (Figure 3D; Supplementary Table S6), the RAS network was more similar to controls than in the case of men. Significant interactions were noted between AT1-AAs and AT2-AAs (r_s_ = 0.425, *p* = 0.001) and between AT1-AAs and MasR-AAs (r_s_ = 0.358, *p* = 0.005), whereas ACE2-AAs remained marginally correlated with AT2-AAs (r_s_ = 0.255, *p* = 0.051) and not significantly associated with MasR-AAs. Additionally, a more robust correlation was observed between AT2-AAs and MasR-AAs (r_s_ = 0.472, *p* < 0.001), suggesting a reinforced coupling between these two protective RAS receptors in the control women as compared with control men.

Overall, the results indicate sex-dependent differences in RAS autoantibody co-regulation in both physiological and pathological states. Notably, PD is associated with the emergence of a more coupled RAS network, particularly pronounced in parkinsonian men, suggesting a potential mechanistic link between RAS-AA dysregulation and sex-specific disease progression.

### 3.5. Age-Dependent Correlation Networks of RAS Autoantibodies in Controls and Parkinson’s Disease Patients

**Figure 4:**
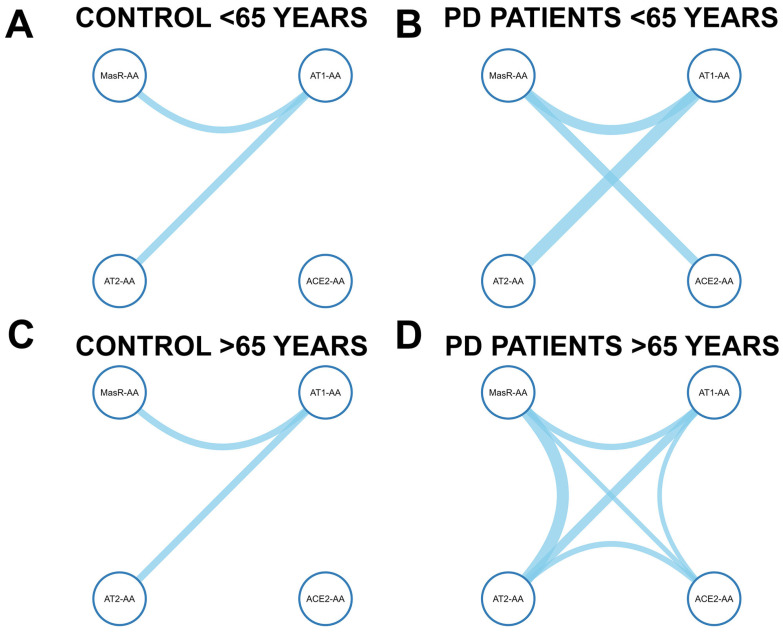
Age-stratified Spearman correlation networks of RAS-related autoantibodies in Parkinson’s disease (PD) patients and healthy controls (<65 *vs.* >65 years). Nodes represent four autoantibodies (AT1-AA, AT2-AA, MasR-AA, ACE2-AA). Edges show significant correlations (*p* < 0.05), with thickness indicating strength (r_s_).

Age-stratified correlation analysis revealed marked differences in the organization of RAS autoantibody networks between younger and older individuals, as well as between PD patients and controls (Figure 4).

In controls under 65 years of age (Figure 4A; Supplementary Table S7), a sparsely connected network was observed. The only significant correlations involved AT1-AAs and AT2-AAs (r_s_ = 0.362, *p* = 0.010), and AT1-AAs and MasR-AAs (r_s_ = 0.319, *p* = 0.029). No correlations involving ACE2-AAs reached statistical significance. In over-65-year controls, following the same pattern as in the under-65-year control group, the network remained sparse (Figure 4C; Supplementary Table S8). Only significant correlations were found between AT1-AAs and AT2-AAs (r_s_ = 0.335, *p* = 0.025), and between AT1-AAs and MasR-AAs (r_s_ = 0.317, *p* = 0.038). ACE2-AAs were not significantly correlated with any of the other markers. In contrast, PD patients under 65 years (Figure 4B; Supplementary Table S9) exhibited a more interconnected RAS network, with AT1-AAs significantly associated with AT2-AAs (r_s_ = 0.593, *p* < 0.001) and MasR-AAs (r_s_ = 0.473, *p* = 0.006), and MasR-AAs significantly associated with ACE2-AAs (r_s_ = 0.427, *p* = 0.015). Although the correlation between AT1-AAs and ACE2-AAs did not reach statistical significance (r_s_ = 0.320, *p* = 0.070), it showed a trend toward positive association. PD patients older than 65 years (Figure 4D; Supplementary Table S10) showed a more integrated and dense correlation network. Significant correlations were observed between AT1-AAs and AT2-AAs (r_s_ = 0.411, *p* < 0.001), AT1-AAs and MasR-AAs (r_s_ = 0.295, *p* = 0.007), as well as between ACE2-AAs and AT2-AAs (r_s_ = 0.270, *p* = 0.013) and MasR-AAs (r_s_ = 0.225, *p* = 0.041). AT1-AA and ACE2-AA correlation, although weaker, was statistically significant (r_s_ = 0.216, *p* = 0.049). Additionally, a significant correlation was observed between AT2-AAs and MasR-AAs (r_s_ = 0.57, *p* < 0.001).

These results suggest that Parkinson’s disease promotes a progressive reinforcement and integration of these RAS-AA networks, particularly evident in older patients.

### 3.6. Immune and Oxidative Crosstalk with the RAS Autoantibody Network in Parkinson’s Disease Patients

**Figure 5:**
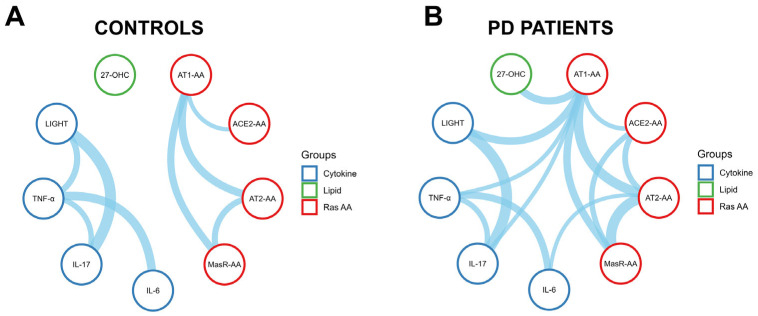
Integrated correlation networks of RAS autoantibodies, cytokines, and oxidative stress markers in healthy controls (**A**) and Parkinson’s disease (PD) patients (**B**). Nodes represent RAS autoantibodies (red), cytokines (blue), and 27-OHC (green). Edges show significant correlations (*p* < 0.05), with thickness indicating strength (r_s_). Controls showed two loosely connected modules (RAS and cytokines), while the PD network was more integrated, with AT1-AA as a central hub linking immune, oxidative, and RAS pathways.

We analyzed the correlation network among RAS-related autoantibodies, pro-inflammatory cytokines, and the lipid oxidation marker 27-hydroxycholesterol (27-OHC). While these systems have been individually implicated in PD, their integrated response remains unclear. A cluster was observed among RAS receptor autoantibodies in controls (Figure 5A; Supplementary Table S11). See Section 3.3 above for details. Regarding inflammatory markers, IL-6 and TNF-α displayed a significant correlation (r_s_ = 0.409, *p* < 0.001). Additionally, IL-17 was significantly correlated with both TNF-α (r_s_ = 0.259, *p* = 0.009) and LIGHT (r_s_ = 0.520, *p* < 0.001), while TNF-α also showed a positive association with LIGHT (r_s_ = 0.294, *p* = 0.003). No significant associations were found between RAS-AAs and inflammatory mediators, and the lipid oxidation marker 27-OHC remained largely disconnected in controls. In contrast, PD patients (Figure 5B; Supplementary Table S12) exhibited a dense and more integrated correlation network. A central cluster emerged, encompassing RAS autoantibodies (AT1-AAs, AT2-AAs, MasR-AAs and ACE2-AAs), pro-inflammatory cytokines (IL-6, IL-17, TNF-α, LIGHT), and 27-OHC. AT1-AAs functioned as a hub node, displaying significant correlations with AT2-AAs (r_s_ = 0.460, *p* < 0.001), MasR-AAs (r_s_ = 0.355, *p* < 0.001), IL-17 (r_s_ = 0.197, *p* = 0.034), TNF-α (r_s_ = 0.202, *p* = 0.030), LIGHT (r_s_ = 0.359, *p* < 0.001), 27-OHC (r_s_ = 0.347, *p* < 0.001), and ACE2 (r_s_ = 0.215, *p* = 0.020). Additional significant associations included those between ACE2-AAs and AT2-AAs (r_s_ = 0.274, *p* = 0.003), ACE2-AAs and MasR-AAs (r_s_ = 0.257, *p* = 0.006), AT2-AAs and MasR-AAs (r_s_ = 0.491, *p* < 0.001), and AT2-AAs and IL-6 (r_s_ = 0.185, *p* = 0.047). Among cytokines, IL-6 correlated with TNF-α (r_s_ = 0.335, *p* < 0.001), and IL-17 was significantly associated with both TNF-α (r_s_ = 0.256, *p* = 0.006) and LIGHT (r_s_ = 0.509, *p* < 0.001). Notably, the increased correlation density among RAS autoantibodies and cytokines points to a systemic inflammatory phenotype in the patient group, potentially contributing to neurodegenerative progression.

### 3.7. Multivariate Analysis Identifies Predictors of PD Status

A binary logistic regression model was employed to assess the association between the analyzed variables and PD status. The model was trained on 80% of the dataset, while the remaining 20% was reserved for evaluating predictive performance. The full model included all candidate biomarkers and covariates. In this model, both AT1-AAs (β = 0.057, *p* = 0.050) and age (β = 0.050, *p* = 0.010) were significantly associated with PD diagnosis, whereas other variables, including MasR-AAs and ACE2-AAs, did not reach statistical significance (Supplementary Table S13). To enhance model parsimony and reduce potential overfitting, a stepwise selection procedure based on the Akaike Information Criterion (AIC) was applied. The resulting reduced model Equation (1) retained AT1-AAs, MasR-AAs, and age as predictors:

Equation (1): Logistic Regression Equation for Group Classification (Patient vs. Control)

(1) $$\log\left[\frac{P\;\left(\mathrm{Group}=\mathrm{Patient}\right)}{1-P\;\left(\mathrm{Group}=\mathrm{Patient}\right)}\right]=\beta_{0}+\beta_{1}\left(AT1-AAs\right)+\beta_{2}\;\left(\mathrm{MasR}-\mathrm{AAs}\right)+\beta_{3}\left(\mathrm{Age}\right)$$

In the reduced model (Supplementary Table S14), AT1-AAs (β = 0.062, *p* = 0.034) and age (β = 0.053, *p* = 0.005) remained statistically significant, while MasR-AAs showed a non-significant trend (β = 0.010, *p* = 0.074). The corresponding Odds Ratios (OR) were 1.063 for AT1-AAs (95% CI: 1.014–1.137) and 1.055 for age (95% CI: 1.017–1.097), indicating a positive association with PD status.

**Figure 6:**
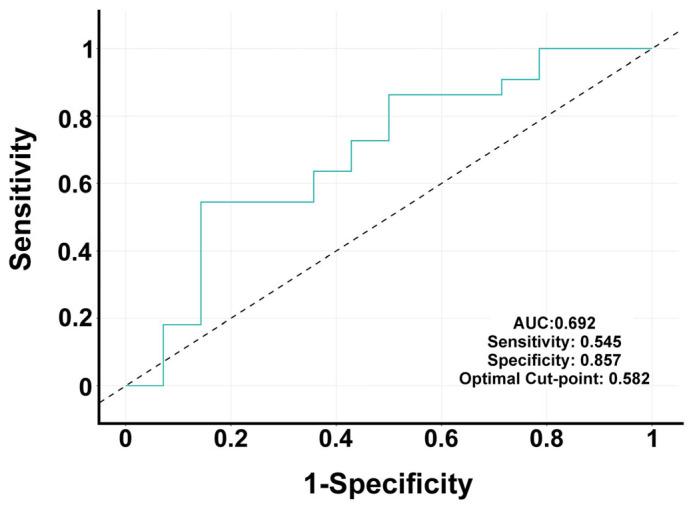
Receiver operating characteristic (ROC) curve evaluating the diagnostic performance of the model for classifying PD patients. The ROC curve illustrates the trade-off between sensitivity and specificity across different threshold values. The area under the curve (AUC) was 0.692, indicating moderate discriminative ability. At the optimal cutoff point of 0.582, the sensitivity was 0.545 and the specificity was 0.857. These results suggest that the marker provides high specificity but limited sensitivity in distinguishing between groups.

Model calibration assessed using the Hosmer–Lemeshow test indicated a good fit (χ^2^ = 1.578, *p* = 0.664). Predictive performance on the test set (20% of the data) showed a sensitivity of 0.545, specificity of 0.857, positive predictive value of 0.857, negative predictive value of 0.545, and overall accuracy of 0.667. The area under the ROC curve (AUC, Figure 6) was 0.692 (95% CI: 0.502–0.881), showing a moderate discriminative ability of the model.

## 4. Discussion

In previous studies, we observed an increase in the levels of pro-oxidative/pro-inflammatory receptor autoantibodies (AT1-AAs and ACE2-AAs) in PD patients. In the present study, we observed that PD patients showed a significant increase in serum levels of the protective MasR-AAs. However, we did not detect any significant increase in the levels of serum AT2-AAs. Furthermore, the autoantibody correlation signature showed some changes in PD patients that revealed a more interconnected cluster of correlations among the levels of RAS receptor autoantibodies (AT1-AAs, AT2-AAs, MasR-AAs, ACE2-AAs).

Dysregulation of GPCR-AAs may promote tissue damage by uncoupling receptors from the endogenous signaling network [24]. Consistent with this, we have recently shown that AT1-AAs induced neurodegeneration in PD models through several mechanisms, such as promoting oxidative stress and neuroinflammation, intraneuronal calcium raising, alpha-synuclein aggregation, and blood–brain barrier disruption, which were mitigated by treatment with AT1 receptor blockers [13,14,19]. AT1-AAs are powerful activators of AT1 receptors (i.e., of the RAS pro-oxidative/inflammatory axis), because they act as AT1 agonists and, particularly, because the binding of AT1-AAs blocks AT1 internalization and stabilizes the AT1 receptor in a permanent activation [35], leading to upregulation AT1 receptor expression and receptor sensitization [36,37,38]. It is known that AT1 internalization after angiotensin-II binding, which is blocked by AT1-AA binding, is a major mechanism for counteracting sustained AT1 receptor activation [39,40].

Conversely, it is also known that tissue damage and inflammation may increase levels of AAs and break down their homeostasis. AA production may be modulated simply by increasing or decreasing receptor expression [26,41], which may occur in response to physiological, and especially pathological, changes such as those observed in PD [2,42,43,44]. Furthermore, pathological and inflammatory processes may increase the formation of neoantigens. The production of AT1-AAs has been associated with the release of inflammation-related cytokines, because administration of IL-6 and TNF-α to animal models induced the production of AT1-AAs [16,45]. Interestingly, the cytokine TNFSF14 (LIGHT), acting through tissue transglutaminase 2 (TG2), plays a major role in AT1-AA generation [36]. TG2-induced modification of AT1 receptors [37,46] led to the formation of neoantigens that induced AT1-AA production [46]. In the present and previous studies, we also observed a significant correlation between AT1-AAs and LIGHT levels in animal models and PD patients [13,14,47].

Inflammatory processes usually induce compensatory anti-inflammatory responses to counteract excessive immune activation [48,49]. Consistent with this, AT1 overactivity induces upregulation of compensatory AT2 and MasR receptors [40,50,51]. In the present study, we observed upregulation of anti-inflammatory MasR-AAs and alteration in correlations between pro-oxidative/inflammatory and anti-oxidative/inflammatory RAS receptor autoantibodies. Increased expression of AT2 and Mas receptors in cells as a consequence of AT1 overactivity and inflammation may lead to an increase in the release of the corresponding antigens and upregulation in levels of agonistic AT2 and MasR autoantibodies. However, no consistent increase in AT2-AA levels was detected in the serum of PD patients in the present study, which may reduce the compensation of the effects of pro-inflammatory AT1-AA. Furthermore, ACE2-AAs play an inhibitory effect on ACE2 activity [20,21], promoting the downregulation of the RAS anti-inflammatory arm by reducing the levels of Ang1-7. The increase in ACE2-AAs observed in PD patients [13], therefore, counteracts the possible beneficial effects of Mas receptor stimulation by MasR-AA observed in the present study. The fact that AT2-AA shows strong correlations with other autoantibodies in PD patients, despite no significant change in its mean concentration between groups, might seem striking at first glance. However, this may reflect a shift in the covariance structure rather than a difference in the mean. This suggests that AT2-AA becomes more functionally integrated into the autoantibody network in the disease context—a phenomenon that does not require an increased concentration, but rather a different pattern of joint variability among autoantibodies. Moreover, it is worth noting that AT2-AA levels are indeed higher in patients, although the difference does not reach statistical significance (*p* = 0.175). In addition, biological factors may also be involved. Consistent with previous studies on different autoantibodies and pathological processes [27,52], we observed differences in the organization of RAS autoantibody networks according to sex and age, both in controls and in PD patients, as revealed by the stratified correlation analyses. PD is associated with the emergence of a tightly coupled RAS network, particularly pronounced in male patients, suggesting a potential mechanistic link between RAS dysregulation and sex-specific disease progression. Aging also affected correlation networks of RAS autoantibodies, which was particularly observed in PD patients. PD promotes a progressive reinforcement and integration of these RAS-AA correlation networks, which is particularly evident in older patients.

Interestingly, we observed differences between PD patients and controls in correlation networks of RAS autoantibodies and major PD-related pro-inflammatory cytokines and 27-OHC. In controls, correlations were observed among cytokines and among RAS autoantibodies. Unlike controls, who exhibited compartmentalized and not significantly connected cytokines and RAS modules, PD patients displayed a higher correlation network linking RAS autoantibodies, pro-inflammatory cytokines, and oxidative stress products such as 27-OHC. This pattern suggests the existence of an integrated RAS-inflammatory-oxidative axis that may reflect systemic pathophysiological processes in PD, and AT1-AAs appear as a central hub within this network, exhibiting statistically significant correlations with both cytokines and lipid markers. This also supports the potential utility of multiplex biomarker panels for disease stratification, progression monitoring, and therapeutic targeting.

These correlations could be expected based on previous experimental data. As indicated above, the production of AT1-AAs has been associated with the release of pro-inflammatory cytokines [16,45], and the cytokine TNFSF14 (LIGHT) plays a major role in the generation of AT1-AAs [36]. Conversely, AT1 activation increases the levels of pro-inflammatory cytokines [13,14] that contribute to the modulation of immune cell invasion, which is, under physiological conditions, necessary to couple with tissue damage [27]. Furthermore, it is known that Mas and AT2 receptor activation downregulates levels of pro-inflammatory cytokines such as IL-6 and others and upregulates anti-inflammatory cytokines such as IL-10 [53,54,55]. The present results also revealed direct and indirect correlations of AT1-AAs and the pro-oxidative lipid marker 27-OHC and IL-17, previously associated with PD progression [28,30,31,56].

It is interesting to remark that the present correlations and RAS AAs were determined in serum samples from both controls and PD patients. However, we observed AT1-AA and ACE2-AA in the cerebrospinal fluid (CSF) of PD models and PD patients in previous studies [13,14], and more recently, we observed AT2-AA and MasR-AA in post-COVID patients, where we observed correlations with levels of serum RAS-AAs [57]. Serum AAs may enter the CSF/brain through a disrupted BBB. The increase in pro-inflammatory cytokines due to the inflammatory process and other factors may contribute to the disruption of the BBB in PD patients; however, high levels of circulating AT1-AA may directly contribute to the disruption of the BBB [58,59,60], increasing BBB permeability to autoantibodies. However, recent studies suggest that activated B cells (activated by neoantigens from RAS receptors in this case) can cross the BBB followed by intrathecal formation of autoantibodies [61,62,63]. Furthermore, recent studies suggest that naive antigen-specific B cell recruitment from the circulation into the dural-associated lymphoid tissues (DALT) is initially required, but that a subsequent germinal center response is held in the DALT independently of circulating input [61]. Our previous studies on AT1-AA and ACE2 in PD patients using the corrected antibody index support the intrathecal formation of autoantibodies [13]. Consistent with the observations in PD patients, we observed the generation of serum RAS-AAs in animal models of PD after inducing dopaminergic degeneration and neuroinflammation after intracerebral injection of neurotoxins (6-OHDA) [13,14]. As described above, dopaminergic degeneration/neuroinflammation modifies AT1 receptors, mediated by inflammatory cytokines and TG2, forming neoantigens that reach the CSF. From the CSF, via the brain lymphatic system, these antigens drain into cervical lymph nodes and activate B cells, inducing antigen-specific B cells and circulating autoantibodies that finally reach the CSF as described above. Interestingly, RAS receptors were also located in extracellular vesicles from brain cells [64,65], which may constitute another possible source for GPCR-AAs production that remains to be studied.

In the present study, multivariate logistic regression analysis identified AT1-AA and age as independent predictors of PD status. The reduced model, which included AT1-AA, MasR-AA, and age, showed good calibration and moderate discriminative performance (AUC = 0.692). Notably, the association between AT1-AA and PD remained significant even after adjustment for age and other variables, suggesting its potential utility as a disease-related immune biomarker. The modest contribution of MasR-AA and the lack of significance for other autoantibodies highlight the specificity of AT1–AA–mediated immune activity in the context of PD. The increase in levels of RAS autoantibodies cannot be a specific marker of PD, as was observed in other inflammation-related processes. However, the observed biomarker patterns, together with a panel of other biomarkers, may provide valuable information for PD progression monitoring and therapeutic targeting. Consistent with this, in a recent study [13], we observed that each unit increase in serum AT1-AA concentration was associated with a 7.4% increase in the odds of PD diagnosis. Furthermore, high levels of AT1-AA may also identify PD patients with higher therapeutic response to AT1 receptor blockers [66].

Several limitations should be acknowledged. First, the cross-sectional design does not allow for causal inferences, and longitudinal studies will be needed to determine whether the observed biomarker patterns preceded or resulted from neurodegeneration. Nevertheless, the current data provide a valuable snapshot of the biological landscape associated with the disease and offer a strong foundation for future hypotheses. Second, the sample size in some stratified subgroups was limited, which may have affected statistical power. However, the main findings were consistently observed across analyses, supporting their robustness. Third, our focus was limited to circulating biomarkers, and their direct relationship with central nervous system alterations remains to be fully elucidated. Despite these limitations, the consistency of network-level patterns and the stability of the multivariate model underscore the relevance and reliability of the present findings.

## 5. Conclusions

Our findings highlight a dysregulated autoantibody network targeting the RAS in PD, characterized by increased levels of pro-oxidative/pro-inflammatory AT1-AAs and ACE2-AAs and protective anti-oxidative/anti-inflammatory MasR-AAs, along with altered correlations among RAS-AAs and cytokines and oxidative stress markers such as 27-OHC. AT1-AAs emerged as a central component of an integrated RAS-AA inflammatory/oxidative network associated with PD, suggesting their potential as disease-related immune biomarkers. This study reveals a disease-specific network of RAS autoantibodies in PD that links immune and oxidative pathways and identifies new biomarker patterns and potential therapeutic targets.

## Data Availability

All figures and data used to support this study are included within this article; further inquiries can be directed to the corresponding authors on reasonable request.

## Supplementary Materials

The following supporting information can be downloaded at: https://www.mdpi.com/article/10.3390/antiox14060706/s1, Figure S1: AT1-AA and ACE2-AA serum levels in controls and PD patients; Table S1: Pairwise Spearman correlation analysis among RAS-related autoantibodies in the entire control group; Table S2: Pairwise Spearman correlation analysis among RAS-related autoantibodies in the full cohort of Parkinson’s disease patients; Table S3: Pairwise Spearman correlation analysis among RAS-related autoantibodies in the control man group; Table S4: Pairwise Spearman correlation analysis among RAS-related autoantibodies in the control woman group; Table S5: Pairwise Spearman correlation analysis among RAS-related autoantibodies in parkinsonian men; Table S6: Pairwise Spearman correlation analysis among RAS-related autoantibodies in parkinsonian women; Table S7: Pairwise Spearman correlation analysis among RAS-related autoantibodies in the younger (<65 years) control group; Table S8: Pairwise Spearman correlation analysis among RAS-related autoantibodies in the older (>65 years) control group; Table S9: Pairwise Spearman correlation analysis among RAS-related autoantibodies in the younger (<65 years) parkinsonian group; Table S10: Pairwise Spearman correlation analysis among RAS-related autoantibodies in the older (>65 years) parkinsonian group; Table S11: Pairwise Spearman correlation analysis among RAS-related autoantibodies, inflammatory cytokines, and 27-hydroxycholesterol in the full control cohort; Table S12: Pairwise Spearman correlation analysis among RAS-related autoantibodies, inflammatory cytokines, and 27-hydroxycholesterol in the full parkinsonian cohort; Table S13: Binary logistic regression analysis of the association between selected variables and Parkinson’s disease status. Full model; Table S14: Binary logistic regression analysis of the association between selected variables and Parkinson’s disease status. Reduced model.

## Abbreviations

The following abbreviations are used in this manuscript:

27-OHC	27-hydroxycholesterol
6-OHDA	6-hydroxydopamine
ACE2	Angiotensin-converting enzyme 2
ACE2-AAs	ACE2 autoantibodies
AIC	Akaike Information Criterion
Ang II	Angiotensin II
Ang1-7	Angiotensin-(1-7)
AUC	Area under curve
AT1R	AT1 receptors
AT2-AAs	AT2 autoantibodies
AT2R	AT2 receptors
AAs	Autoantibodies
AT1-AA	Autoantibodies for AT1 receptors
BBB	Blood–brain barrier
DALT	Dural-associated lymphoid tissues
ELISA	Enzyme-linked immunosorbent assay
4PL	Four-parameter logistic
GPCR	G protein-coupled receptors
CEIm-G	Galician Drug Research Ethics Committee
CSF	Cerebrospinal fluid
IL-17	Interleukin-17
IL-6	Interleukin-6
IQR	Interquartile ranges
MasR	Mas receptors
MasR-AAs	MasR autoantibodies
PD	Parkinson’s disease
ROC	Receiver operating characteristic
RAS	Renin–angiotensin system
TG2	Transglutaminase 2
TNFSF14, LIGHT	Tumor necrosis factor superfamily member 14
TNF-α	Tumor necrosis factor-alpha

## References

[1] Jackson L., Eldahshan W., Fagan S.C., Ergul A. (2018). Within the Brain: The Renin Angiotensin System. Int. J. Mol. Sci..

[2] Labandeira-Garcia J.L., Labandeira C.M., Guerra M.J., Rodriguez-Perez A.I. (2024). The role of the brain renin-angiotensin system in Parkinson’s disease. Transl. Neurodegener..

[3] Costa-Besada M.A., Valenzuela R., Garrido-Gil P., Villar-Cheda B., Parga J.A., Lanciego J.L., Labandeira-Garcia J.L. (2018). Paracrine and Intracrine Angiotensin 1-7/Mas Receptor Axis in the Substantia Nigra of Rodents, Monkeys, and Humans. Mol. Neurobiol..

[4] Valenzuela R., Rodriguez-Perez A.I., Costa-Besada M.A., Rivas-Santisteban R., Garrido-Gil P., Lopez-Lopez A., Navarro G., Lanciego J.L., Franco R., Labandeira-Garcia J.L. (2021). An ACE2/Mas-related receptor MrgE axis in dopaminergic neuron mitochondria. Redox Biol..

[5] Bild W., Vasincu A., Rusu R.N., Ababei D.C., Stana A.B., Stanciu G.D., Savu B., Bild V. (2022). Impact of the Renin-Angiotensin System on the Pathogeny and Pharmacotherapeutics of Neurodegenerative Diseases. Biomolecules.

[6] Kamath T., Abdulraouf A., Burris S.J., Langlieb J., Gazestani V., Nadaf N.M., Balderrama K., Vanderburg C., Macosko E.Z. (2022). Single-cell genomic profiling of human dopamine neurons identifies a population that selectively degenerates in Parkinson’s disease. Nat. Neurosci..

[7] Lee A.J., Kim C., Park S., Joo J., Choi B., Yang D., Jun K., Eom J., Lee S.J., Chung S.J. (2023). Characterization of altered molecular mechanisms in Parkinson’s disease through cell type-resolved multiomics analyses. Sci. Adv..

[8] Martirosyan A., Ansari R., Pestana F., Hebestreit K., Gasparyan H., Aleksanyan R., Hnatova S., Poovathingal S., Marneffe C., Thal D.R. (2024). Unravelling cell type-specific responses to Parkinson’s Disease at single cell resolution. Mol. Neurodegener..

[9] Jo Y., Kim S., Ye B.S., Lee E., Yu Y.M. (2022). Protective Effect of Renin-Angiotensin System Inhibitors on Parkinson’s Disease: A Nationwide Cohort Study. Front. Pharmacol..

[10] Lin H.C., Tseng Y.F., Shen A.L., Chao J.C., Hsu C.Y., Lin H.L. (2022). Association of Angiotensin Receptor Blockers with Incident Parkinson Disease in Patients with Hypertension: A Retrospective Cohort Study. Am. J. Med..

[11] Romanowska J., Bjornevik K., Cortese M., Tuominen J.A., Solheim M., Abolpour Mofrad A., Igland J., Scherzer C.R., Riise T. (2023). Association Between Use of Any of the Drugs Prescribed in Norway and the Subsequent Risk of Parkinson Disease: A Drug-wide Association Study. Neurology.

[12] Tansey M.G., Wallings R.L., Houser M.C., Herrick M.K., Keating C.E., Joers V. (2022). Inflammation and immune dysfunction in Parkinson disease. Nat. Rev. Immunol..

[13] Labandeira C.M., Pedrosa M.A., Quijano A., Valenzuela R., Garrido-Gil P., Sanchez-Andrade M., Suarez-Quintanilla J.A., Rodriguez-Perez A.I., Labandeira-Garcia J.L. (2022). Angiotensin type-1 receptor and ACE2 autoantibodies in Parkinson’s disease. NPJ Park. Dis..

[14] Pedrosa M.A., Labandeira C.M., Valenzuela R., Quijano A., Sanchez-Andrade M., Suarez-Quintanilla J.A., Lanciego J.L., Labandeira-Garcia J.L., Rodriguez-Perez A.I. (2023). AT1 receptor autoantibodies mediate effects of metabolic syndrome on dopaminergic vulnerability. Brain Behav. Immun..

[15] Giral M., Foucher Y., Dufay A., Duong Van Huyen J.P., Renaudin K., Moreau A., Philippe A., Hegner B., Dechend R., Heidecke H. (2013). Pretransplant sensitization against angiotensin II type 1 receptor is a risk factor for acute rejection and graft loss. Am. J. Transplant..

[16] Lamarca B., Speed J., Ray L.F., Cockrell K., Wallukat G., Dechend R., Granger J. (2011). Hypertension in response to IL-6 during pregnancy: Role of AT1-receptor activation. Int. J. Interferon Cytokine Mediat. Res..

[17] Riemekasten G., Philippe A., Nather M., Slowinski T., Muller D.N., Heidecke H., Matucci-Cerinic M., Czirjak L., Lukitsch I., Becker M. (2011). Involvement of functional autoantibodies against vascular receptors in systemic sclerosis. Ann. Rheum. Dis..

[18] Wallukat G., Homuth V., Fischer T., Lindschau C., Horstkamp B., Jupner A., Baur E., Nissen E., Vetter K., Neichel D. (1999). Patients with preeclampsia develop agonistic autoantibodies against the angiotensin AT1 receptor. J. Clin. Investig..

[19] Lage L., Rodriguez-Perez A.I., Labandeira-Garcia J.L., Dominguez-Meijide A. (2024). Angiotensin type-1 receptor autoantibodies promote alpha-synuclein aggregation in dopaminergic neurons. Front. Immunol..

[20] Arthur J.M., Forrest J.C., Boehme K.W., Kennedy J.L., Owens S., Herzog C., Liu J., Harville T.O. (2021). Development of ACE2 autoantibodies after SARS-CoV-2 infection. PLoS ONE.

[21] Takahashi Y., Haga S., Ishizaka Y., Mimori A. (2010). Autoantibodies to angiotensin-converting enzyme 2 in patients with connective tissue diseases. Arthritis Res. Ther..

[22] Wallukat G., Wernike K., Bachamanda Somesh D., Mettenleiter T.C., Muller J. (2023). Animals Experimentally Infected with SARS-CoV-2 Generate Functional Autoantibodies against G-Protein-Coupled Receptors. Biomedicines.

[23] Liles C., Li H., Veitla V., Liles J.T., Murphy T.A., Cunningham M.W., Yu X., Kem D.C. (2015). AT2R autoantibodies block angiotensin II and AT1R autoantibody-induced vasoconstriction. Hypertension.

[24] Skiba M.A., Kruse A.C. (2021). Autoantibodies as Endogenous Modulators of GPCR Signaling. Trends Pharmacol. Sci..

[25] Cabral-Marques O., Carvalho-Marques A.H., Schimke L.F., Heidecke H., Riemekasten G. (2019). Loss of balance in normal GPCR-mediated cell trafficking. Front. Biosci. (Landmark Ed.).

[26] Cabral-Marques O., Marques A., Giil L.M., De Vito R., Rademacher J., Gunther J., Lange T., Humrich J.Y., Klapa S., Schinke S. (2018). GPCR-specific autoantibody signatures are associated with physiological and pathological immune homeostasis. Nat. Commun..

[27] Riemekasten G., Petersen F., Heidecke H. (2020). What Makes Antibodies Against G Protein-Coupled Receptors so Special? A Novel Concept to Understand Chronic Diseases. Front. Immunol..

[28] Dai L., Wang J., Zhang X., Yan M., Zhou L., Zhang G., Meng L., Chen L., Cao X., Zhang Z. (2023). 27-Hydroxycholesterol Drives the Spread of alpha-Synuclein Pathology in Parkinson’s Disease. Mov. Disord..

[29] Durrenberger P.F., Grunblatt E., Fernando F.S., Monoranu C.M., Evans J., Riederer P., Reynolds R., Dexter D.T. (2012). Inflammatory Pathways in Parkinson’s Disease; A BNE Microarray Study. Park. Dis..

[30] Karpenko M.N., Vasilishina A.A., Gromova E.A., Muruzheva Z.M., Miliukhina I.V., Bernadotte A. (2018). Interleukin-1beta, interleukin-1 receptor antagonist, interleukin-6, interleukin-10, and tumor necrosis factor-alpha levels in CSF and serum in relation to the clinical diversity of Parkinson’s disease. Cell Immunol..

[31] Liu Z., Qiu A.W., Huang Y., Yang Y., Chen J.N., Gu T.T., Cao B.B., Qiu Y.H., Peng Y.P. (2019). IL-17A exacerbates neuroinflammation and neurodegeneration by activating microglia in rodent models of Parkinson’s disease. Brain Behav. Immun..

[32] Hughes A.J., Daniel S.E., Kilford L., Lees A.J. (1992). Accuracy of clinical diagnosis of idiopathic Parkinson’s disease: A clinico-pathological study of 100 cases. J. Neurol. Neurosurg. Psychiatry.

[33] Cohen J. (1988). Statistical Power Analysis for the Behavioral Sciences.

[34] R Core Team R: A Language and Environment for Statistical Computing. https://www.R-project.org/.

[35] Bian J., Lei J., Yin X., Wang P., Wu Y., Yang X., Wang L., Zhang S., Liu H., Fu M.L.X. (2019). Limited AT1 Receptor Internalization Is a Novel Mechanism Underlying Sustained Vasoconstriction Induced by AT1 Receptor Autoantibody from Preeclampsia. J. Am. Heart Assoc..

[36] Liu C., Luo R., Elliott S.E., Wang W., Parchim N.F., Iriyama T., Daugherty P.S., Blackwell S.C., Sibai B.M., Kellems R.E. (2015). Elevated Transglutaminase Activity Triggers Angiotensin Receptor Activating Autoantibody Production and Pathophysiology of Preeclampsia. J. Am. Heart Assoc..

[37] Liu C., Luo R., Wang W., Peng Z., Johnson G.V.W., Kellems R.E., Xia Y. (2019). Tissue Transglutaminase-Mediated AT1 Receptor Sensitization Underlies Pro-inflammatory Cytokine LIGHT-Induced Hypertension. Am. J. Hypertens..

[38] Liu C., Wang W., Parchim N., Irani R.A., Blackwell S.C., Sibai B., Jin J., Kellems R.E., Xia Y. (2014). Tissue transglutaminase contributes to the pathogenesis of preeclampsia and stabilizes placental angiotensin receptor type 1 by ubiquitination-preventing isopeptide modification. Hypertension.

[39] Hein L., Meinel L., Pratt R.E., Dzau V.J., Kobilka B.K. (1997). Intracellular trafficking of angiotensin II and its AT1 and AT2 receptors: Evidence for selective sorting of receptor and ligand. Mol. Endocrinol..

[40] Villar-Cheda B., Costa-Besada M.A., Valenzuela R., Perez-Costas E., Melendez-Ferro M., Labandeira-Garcia J.L. (2017). The intracellular angiotensin system buffers deleterious effects of the extracellular paracrine system. Cell Death Dis..

[41] Kotas M.E., Medzhitov R. (2015). Homeostasis, inflammation, and disease susceptibility. Cell.

[42] Labandeira-Garcia J.L., Rodriguez-Perez A.I., Valenzuela R., Costa-Besada M.A., Guerra M.J. (2016). Menopause and Parkinson’s disease. Interaction between estrogens and brain renin-angiotensin system in dopaminergic degeneration. Front. Neuroendocrinol..

[43] Villar-Cheda B., Dominguez-Meijide A., Valenzuela R., Granado N., Moratalla R., Labandeira-Garcia J.L. (2014). Aging-related dysregulation of dopamine and angiotensin receptor interaction. Neurobiol. Aging.

[44] Villar-Cheda B., Rodriguez-Pallares J., Valenzuela R., Munoz A., Guerra M.J., Baltatu O.C., Labandeira-Garcia J.L. (2010). Nigral and striatal regulation of angiotensin receptor expression by dopamine and angiotensin in rodents: Implications for progression of Parkinson’s disease. Eur. J. Neurosci..

[45] Irani R.A., Zhang Y., Zhou C.C., Blackwell S.C., Hicks M.J., Ramin S.M., Kellems R.E., Xia Y. (2010). Autoantibody-mediated angiotensin receptor activation contributes to preeclampsia through tumor necrosis factor-alpha signaling. Hypertension.

[46] Liu C., Kellems R.E., Xia Y. (2017). Inflammation, Autoimmunity, and Hypertension: The Essential Role of Tissue Transglutaminase. Am. J. Hypertens..

[47] Labandeira C.M., Pedrosa M.A., Suarez-Quintanilla J.A., Cortes-Ayaso M., Labandeira-Garcia J.L., Rodriguez-Perez A.I. (2022). Angiotensin System Autoantibodies Correlate with Routine Prognostic Indicators for COVID-19 Severity. Front. Med..

[48] Debnath M., Berk M., Maes M. (2021). Translational evidence for the Inflammatory Response System (IRS)/Compensatory Immune Response System (CIRS) and neuroprogression theory of major depression. Prog. Neuropsychopharmacol. Biol. Psychiatry.

[49] Woiciechowsky C., Schoning B., Lanksch W.R., Volk H.D., Docke W.D. (1999). Mechanisms of brain-mediated systemic anti-inflammatory syndrome causing immunodepression. J. Mol. Med..

[50] Rodriguez-Perez A.I., Garrido-Gil P., Pedrosa M.A., Garcia-Garrote M., Valenzuela R., Navarro G., Franco R., Labandeira-Garcia J.L. (2020). Angiotensin type 2 receptors: Role in aging and neuroinflammation in the substantia nigra. Brain Behav. Immun..

[51] Sun Y., Li Y., Wang M., Yue M., Bai L., Bian J., Hao W., Sun J., Zhang S., Liu H. (2020). Increased AT(2)R expression is induced by AT(1)R autoantibody via two axes, Klf-5/IRF-1 and circErbB4/miR-29a-5p, to promote VSMC migration. Cell Death Dis..

[52] Cabral-Marques O., Riemekasten G. (2017). Functional autoantibodies targeting G protein-coupled receptors in rheumatic diseases. Nat. Rev. Rheumatol..

[53] Dhande I., Ali Q., Hussain T. (2013). Proximal tubule angiotensin AT2 receptors mediate an anti-inflammatory response via interleukin-10: Role in renoprotection in obese rats. Hypertension.

[54] Garrido-Gil P., Pedrosa M.A., Garcia-Garrote M., Pequeno-Valtierra A., Rodriguez-Castro J., Garcia-Souto D., Rodriguez-Perez A.I., Labandeira-Garcia J.L. (2022). Microglial angiotensin type 2 receptors mediate sex-specific expression of inflammatory cytokines independently of circulating estrogen. Glia.

[55] Quijano A., Rodriguez-Perez A.I., Costa-Besada M.A., Lopez-Lopez A., Guerra M.J., Labandeira-Garcia J.L., Valenzuela R. (2024). Modulation of Mitochondrial Dynamics by the Angiotensin System in Dopaminergic Neurons and Microglia. Aging Dis..

[56] Sommer A., Marxreiter F., Krach F., Fadler T., Grosch J., Maroni M., Graef D., Eberhardt E., Riemenschneider M.J., Yeo G.W. (2018). Th17 Lymphocytes Induce Neuronal Cell Death in a Human iPSC-Based Model of Parkinson’s Disease. Cell Stem Cell.

[57] Rodriguez-Perez A.I., Serrano-Heras G., Labandeira C.M., Camacho-Meno L., Castro-Robles B., Suarez-Quintanilla J.A., Munoz-Lopez M., Piqueras-Landete P., Guerra M.J., Segura T. (2025). Serum angiotensin type-1 receptor autoantibodies and neurofilament light chain as markers of neuroaxonal damage in post-COVID patients. Front. Immunol..

[58] Fleegal-DeMotta M.A., Doghu S., Banks W.A. (2009). Angiotensin II modulates BBB permeability via activation of the AT(1) receptor in brain endothelial cells. J. Cereb. Blood Flow Metab..

[59] Setiadi A., Korim W.S., Elsaafien K., Yao S.T. (2018). The role of the blood-brain barrier in hypertension. Exp. Physiol..

[60] Yasar S., Moored K.D., Adam A., Zabel F., Chuang Y.F., Varma V.R., Carlson M.C. (2020). Angiotensin II Blood Levels Are Associated with Smaller Hippocampal and Cortical Volumes in Cognitively Normal Older Adults. J. Alzheimers Dis..

[61] Fitzpatrick Z., Ghabdan Zanluqui N., Rosenblum J.S., Tuong Z.K., Lee C.Y.C., Chandrashekhar V., Negro-Demontel M.L., Stewart A.P., Posner D.A., Buckley M. (2024). Venous-plexus-associated lymphoid hubs support meningeal humoral immunity. Nature.

[62] Negi N., Das B.K. (2020). Decoding intrathecal immunoglobulins and B cells in the CNS: Their synthesis, function, and regulation. Int. Rev. Immunol..

[63] Sun B., Ramberger M., O’Connor K.C., Bashford-Rogers R.J.M., Irani S.R. (2020). The B cell immunobiology that underlies CNS autoantibody-mediated diseases. Nat. Rev. Neurol..

[64] Nager A.R., Goldstein J.S., Herranz-Perez V., Portran D., Ye F., Garcia-Verdugo J.M., Nachury M.V. (2017). An Actin Network Dispatches Ciliary GPCRs into Extracellular Vesicles to Modulate Signaling. Cell.

[65] Pedrosa M.A., Labandeira C.M., Lago-Baameiro N., Valenzuela R., Pardo M., Labandeira-Garcia J.L., Rodriguez-Perez A.I. (2023). Extracellular Vesicles and Their Renin-Angiotensin Cargo as a Link between Metabolic Syndrome and Parkinson’s Disease. Antioxidants.

[66] Gonzalez-Robles C., Athauda D., Barber T.R., Barker R.A., Dexter D.T., Duty S., Ellis-Doyle R., Gandhi S., Handley J., Jabbari E. (2025). Treatment Selection and Prioritization for the EJS ACT-PD MAMS Trial Platform. Mov. Disord..

