# Supplementary material for: Renin–Angiotensin System Autoantibody Network in Parkinson’s Disease Patients
Source: Antioxidants (Basel). 2025 Jun 10;14(6):706. doi: 10.3390/antiox14060706 (PMC12189439; doi:10.3390/antiox14060706)
Supplement: Supplementary file 1 [file antioxidants-14-00706-s001.zip › antioxidants-3676553-supplementary.pdf]

Article

Supplementary Materials:

Renin-Angiotensin System Autoantibody network in Parkinson's Disease patients

Carmen M. Labandeira[1,+], Laura Camacho-Meño [2,+], Paula Aracil-Pastor [2], Juan A. Suárez-Quintanilla[3], Jose L. Labandeira-García[2,4,5*] and Ana I. Rodríguez-Pérez [2,4,5,*]

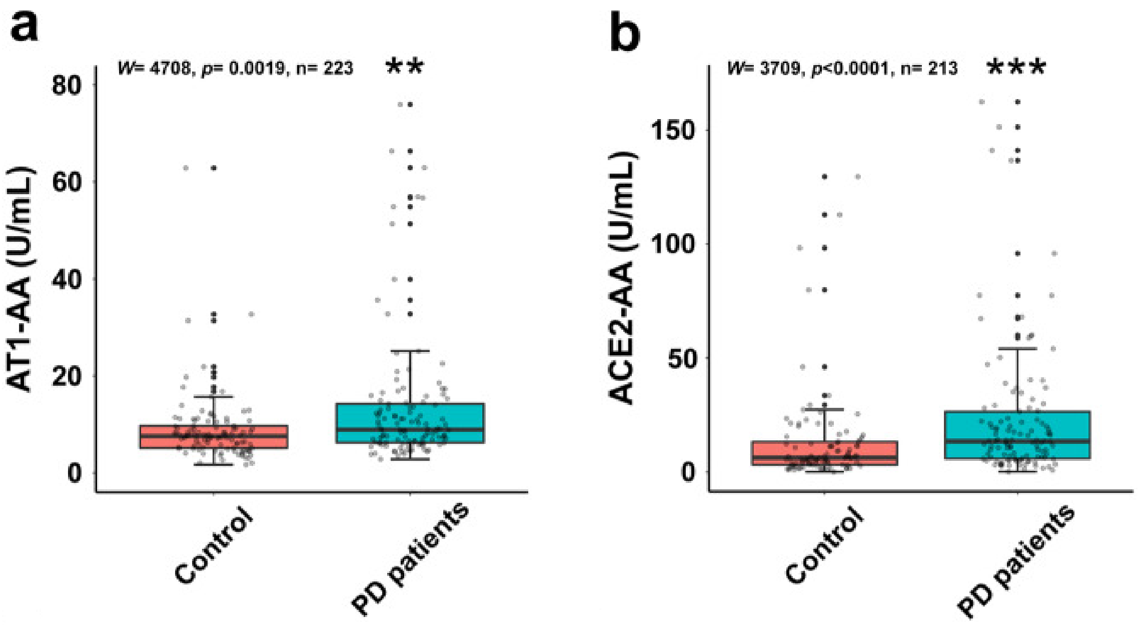

Figure S1. AT1-AA **(a)** and ACE2-AA **(b)** serum levels in controls and PD patients. Wilcoxon–Mann–Whitney test analysis also revealed significantly elevated serum concentrations of both AT1-AA ($P = 0.0019$; $W = 4708$) and ACE2-AA ($P < 0.0001$; $W = 3709$) in PD patients compared to controls. Median serum concentrations of AT1-AA were 7.556 [IQR: 5.109–9.699] U/mL in the control group and 8.923 [IQR: 6.271–14.240] U/mL in PD patients. For ACE2-AA, median serum levels were 6.305 [IQR: 3.265–13.349] U/mL in controls and 13.519 [IQR: 5.974–26.576] U/mL in the PD group. $^{**}P < 0.01$; $^{***}P < 0.001$. From [13].

Table S1. Pairwise Spearman correlation analysis among RAS-related autoantibodies in the entire control group

Variable 1	Variable 2	Correlation Coefficient	95% CI Lower Limit	95% CI Upper Limit	S Statistic	p-value	N
AT1-AAs	ACE2-AAs	0.204	-0.002	0.394	117,378.000	0.046	96
AT1-AAs	AT2-AAs	0.367	0.173	0.533	93,385.234	<0.001	96
AT1-AAs	MasR-AAs	0.341	0.139	0.515	82,778.456	0.001	91
ACE2-AAs	AT2-AAs	0.19	-0.018	0.382	115,742.826	0.065	95
ACE2-AAs	MasR-AAs	0.11	-0.105	0.316	108,098.071	0.301	90
AT2_AAs	MasR-AAs	0.288	0.08	0.472	86,479.830	0.006	90

Spearman correlation coefficients with 95% confidence intervals, S statistics, and associated p-values for pairwise associations among RAS-related autoantibodies in the total control group. N indicates the number of valid pairwise observations. AT1-AAs: Autoantibodies for AT1; AT2-AAs: Autoantibodies for AT2; ACE2-AAs: Autoantibodies for ACE2; MasR-AAs: Autoantibodies for MasR autoantibodies. Statistically significant *p*-values are denoted in red.

Table S2. Pairwise Spearman correlation analysis among RAS-related autoantibodies in the full cohort of Parkinson's disease patients.

Variable 1	Variable 2	Correlation Coefficient	95% CI Lower Limit	95% CI Upper Limit	S Statistic	p-value	N
AT1-AAs	ACE2-AAs	0.215	0.029	0.386	209,619.893	0.02	117
AT1-AAs	AT2-AAs	0.46	0.298	0.597	136,781.770	<0.001	115
AT1-AAs	MasR-AAs	0.355	0.178	0.509	163,581.440	<0.001	115
ACE2-AAs	AT2-AAs	0.274	0.091	0.44	183,904.000	0.003	115
ACE2-AAs	MasR-AAs	0.257	0.072	0.425	188,402.380	0.006	115
AT2_AAs	MasR-AAs	0.491	0.333	0.622	129,000.161	<0.001	115

Spearman correlation coefficients with 95% confidence intervals, S statistics, and associated p-values for pairwise associations among RAS-related autoantibodies in the total parkinsonian group. N indicates the number of valid pairwise observations. AT1-AAs: Autoantibodies for AT1; AT2-AAs: Autoantibodies for AT2; ACE2-AAs: Autoantibodies for ACE2; MasR-AAs: Autoantibodies for MasR autoantibodies. Statistically significant *p*-values are denoted in red.

Table S3. Pairwise Spearman correlation analysis among RAS-related autoantibodies in the control man group.

Variable 1	Variable 2	Correlation Coefficient	95% CI Lower Limit	95% CI Upper Limit	S Statistic	p-value	N
AT1-AAs	ACE2-AAs	0.017	-0.286	0.317	14,916.000	0.91	45
AT1-AAs	AT2-AAs	0.396	0.111	0.621	9,794.039	0.006	46
AT1-AAs	MasR-AAs	0.257	-0.053	0.521	10,547.487	0.093	44
ACE2-AAs	AT2-AAs	0.149	-0.16	0.431	12,915.254	0.328	45
ACE2-AAs	MasR-AAs	0.144	-0.172	0.434	11,333.928	0.356	43
AT2_AAs	MasR-AAs	0.126	-0.186	0.415	12,397.495	0.414	44

Spearman correlation coefficients with 95% confidence intervals, S statistics, and associated p-values for pairwise associations among RAS-related autoantibodies in the man control group. N indicates the number of valid pairwise observations. AT1-AAs: Autoantibodies for AT1; AT2-AAs: Autoantibodies for AT2; ACE2-AAs: Autoantibodies for ACE2; MasR-AAs: Autoantibodies for MasR autoantibodies. Statistically significant *p*-values are denoted in red.

Table S4. Pairwise Spearman correlation analysis among RAS-related autoantibodies in the control woman group

Variable 1	Variable 2	Correlation Coefficient	95% CI Lower Limit	95% CI Upper Limit	S Statistic	p-value	N
AT1-AAs	ACE2-AAs	0.248	-0.038	0.496	16,626.000	0.08	51
AT1-AAs	AT2-AAs	0.28	-0.007	0.524	15,000.241	0.049	50
AT1-AAs	MasR-AAs	0.337	0.047	0.575	11,459.312	0.02	47
ACE2-AAs	AT2-AAs	0.2	-0.091	0.46	16,655.583	0.163	50
ACE2-AAs	MasR-AAs	0.04	-0.258	0.331	16,599.799	0.788	47
AT2_AAs	MasR-AAs	0.488	0.222	0.686	8,305.683	0.001	46

Spearman correlation coefficients with 95% confidence intervals, S statistics, and associated p-values for pairwise associations among RAS-related autoantibodies in the women group. N indicates the number of valid pairwise observations. AT1-AAs: Autoantibodies for AT1; AT2-AAs: Autoantibodies for AT2; ACE2-AAs: Autoantibodies for ACE2; MasR-AAs: Autoantibodies for MasR autoantibodies. Statistically significant *p*-values are denoted in red.

Table S5. Pairwise Spearman correlation analysis among RAS-related autoantibodies in parkinsonian men.

Variable 1	Variable 2	Correlation Coefficient	95% CI Lower Limit	95% CI Upper Limit	S Statistic	p-value	N
AT1-AAs	ACE2-AAs	0.257	-0.012	0.491	22,936.000	0.054	57
AT1-AAs	AT2-AAs	0.494	0.258	0.674	14,820.000	<0.001	56
AT1-AAs	MasR-AAs	0.37	0.11	0.582	18,441.260	0.005	56
ACE2-AAs	AT2-AAs	0.317	0.052	0.541	19,970.000	0.017	56
ACE2-AAs	MasR-AAs	0.352	0.09	0.568	18,955.296	0.008	56
AT2_AAs	MasR-AAs	0.492	0.255	0.673	14,875.017	<0.001	56

Spearman correlation coefficients with 95% confidence intervals, S statistics, and associated p-values for pairwise associations among RAS-related autoantibodies in parkinsonian men. N indicates the number of valid pairwise observations. AT1-AAs: Autoantibodies for AT1; AT2-AAs: Autoantibodies for AT2; ACE2-AAs: Autoantibodies for ACE2; MasR-AAs: Autoantibodies for MasR autoantibodies. Statistically significant *p*-values are denoted in red.

Table S6. Pairwise Spearman correlation analysis among RAS-related autoantibodies in parkinsonian women.

Variable 1	Variable 2	Correlation Coefficient	95% CI Lower Limit	95% CI Upper Limit	S Statistic	p-value	N
AT1-AAs	ACE2-AAs	0.183	-0.082	0.424	29,394.000	0.161	60
AT1-AAs	AT2-AAs	0.425	0.182	0.619	19,690.000	0.001	59
AT1-AAs	MasR-AAs	0.358	0.105	0.568	21,959.283	0.005	59
ACE2-AAs	AT2-AAs	0.255	-0.009	0.486	25,496.000	0.051	59
ACE2-AAs	MasR-AAs	0.201	-0.066	0.441	27,353.599	0.128	59
AT2_AAs	MasR-AAs	0.472	0.238	0.654	18,079.057	<0.001	59

Spearman correlation coefficients with 95% confidence intervals, S statistics, and associated p-values for pairwise associations among RAS-related autoantibodies in parkinsonian women. N indicates the number of valid pairwise observations. AT1-AAs: Autoantibodies for AT1; AT2-AAs: Autoantibodies for AT2; ACE2-AAs: Autoantibodies for ACE2; MasR-AAs: Autoantibodies for MasR autoantibodies. Statistically significant *p*-values are denoted in red.

Table S7. Pairwise Spearman correlation analysis among RAS-related autoantibodies in the younger (<65 years) control group

Variable 1	Variable 2	Correlation Coefficient	95% CI Lower Limit	95% CI Upper Limit	S Statistic	p-value	N
AT1-AAs	ACE2-AAs	0.127	-0.165	0.399	18,178.000	0.379	50
AT1-AAs	AT2-AAs	0.362	0.084	0.587	13,296.192	0.01	50
AT1-AAs	MasR-AAs	0.319	0.026	0.561	11,781.362	0.029	47
ACE2-AAs	AT2-AAs	0.104	-0.187	0.379	18,649.478	0.47	50
ACE2-AAs	MasR-AAs	0.124	-0.178	0.404	15,155.753	0.407	47
AT2_AAs	MasR-AAs	0.246	-0.053	0.505	13,033.384	0.095	47

Spearman correlation coefficients with 95% confidence intervals, S statistics, and associated p-values for pairwise associations among RAS-related autoantibodies in the younger (<65 years) control group. N indicates the number of valid pairwise observations. AT1-AAs: Autoantibodies for AT1; AT2-AAs: Autoantibodies for AT2; ACE2-AAs: Autoantibodies for ACE2; MasR-AAs: Autoantibodies for MasR autoantibodies. Statistically significant *p*-values are denoted in red.

Table S8. Pairwise Spearman correlation analysis among RAS-related autoantibodies in the older (>65 years) control group

Variable 1	Variable 2	Correlation Coefficient	95% CI Lower Limit	95% CI Upper Limit	S Statistic	p-value	N
AT1-AAs	ACE2-AAs	0.249	-0.057	0.512	11,406.000	0.1	45
AT1-AAs	AT2-AAs	0.335	0.037	0.578	10,101.889	0.025	45
AT1-AAs	MasR-AAs	0.317	0.009	0.57	9,046.415	0.038	43
ACE2-AAs	AT2-AAs	0.262	-0.047	0.525	10,474.988	0.086	44
ACE2-AAs	MasR-AAs	0.069	-0.249	0.373	11,489.862	0.664	42
AT2_AAs	MasR-AAs	0.285	-0.03	0.549	8,819.077	0.067	42

Spearman correlation coefficients with 95% confidence intervals, S statistics, and associated p-values for pairwise associations among RAS-related autoantibodies in the older (>65 years) control group. N indicates the number of valid pairwise observations. AT1-AAs: Autoantibodies for AT1; AT2-AAs: Autoantibodies for AT2; ACE2-AAs: Autoantibodies for ACE2; MasR-AAs: Autoantibodies for MasR autoantibodies. Statistically significant *p*-values are denoted in red.

Table S9. Pairwise Spearman correlation analysis among RAS-related autoantibodies in the younger (<65 years) parkinsonian group

Variable 1	Variable 2	Correlation Coefficient	95% CI Lower Limit	95% CI Upper Limit	S Statistic	p-value	N
AT1-AAs	ACE2-AAs	0.32	-0.037	0.604	4,072.000	0.07	33
AT1-AAs	AT2-AAs	0.593	0.298	0.785	2,220.000	<0.001	32
AT1-AAs	MasR-AAs	0.473	0.138	0.71	2,878.000	0.006	32
ACE2-AAs	AT2-AAs	0.324	-0.038	0.611	3,686.000	0.07	32
ACE2-AAs	MasR-AAs	0.427	0.082	0.681	3,124.000	0.015	32
AT2_AAs	MasR-AAs	0.245	-0.124	0.555	4,118.000	0.176	32

Spearman correlation coefficients with 95% confidence intervals, S statistics, and associated p-values for pairwise associations among RAS-related autoantibodies in the younger (<65 years) parkinsonian group. N indicates the number of valid pairwise observations. AT1-AAs: Autoantibodies for AT1; AT2-AAs: Autoantibodies for AT2; ACE2-AAs: Autoantibodies for ACE2; MasR-AAs: Autoantibodies for MasR autoantibodies. Statistically significant *p*-values are denoted in red.

Table S10. Pairwise Spearman correlation analysis among RAS-related autoantibodies in the older (>65 years) parkinsonian group

Variable 1	Variable 2	Correlation Coefficient	95% CI Lower Limit	95% CI Upper Limit	S Statistic	p-value	N
AT1-AAs	ACE2-AAs	0.216	-0.005	0.416	77,482.000	0.049	84
AT1-AAs	AT2-AAs	0.411	0.209	0.58	56,084.000	<0.001	83
AT1-AAs	MasR-AAs	0.295	0.078	0.485	67,195.693	0.007	83
ACE2-AAs	AT2-AAs	0.27	0.052	0.464	69,516.000	0.013	83
ACE2-AAs	MasR-AAs	0.225	0.003	0.426	73,843.949	0.041	83
AT2_AAs	MasR-AAs	0.57	-0.005	0.416	77,482.000	<0.001	84

Spearman correlation coefficients with 95% confidence intervals, S statistics, and associated p-values for pairwise associations among RAS-related autoantibodies in the older (>65 years) parkinsonian group. N indicates the number of valid pairwise observations. AT1-AAs: Autoantibodies for AT1; AT2-AAs: Autoantibodies for AT2; ACE2-AAs: Autoantibodies for ACE2; MasR-AAs: Autoantibodies for MasR autoantibodies. Statistically significant *p*-values are denoted in red.

Table S11. Pairwise Spearman correlation analysis among RAS-related autoantibodies, inflammatory cytokines, and 27-hydroxycholesterol in the full control cohort.

Variable 1	Variable 2	Correlation Coefficient	95% CI Lower Limit	95% CI Upper Limit	S Statistic	p-value	N
AT1-AAs	IL-6	0.075	-0.127	0.271	163,610.829	0.455	102
AT1-AAs	IL-17	0.049	-0.153	0.248	163,246.556	0.625	101
AT1-AAs	TNF-α	0.097	-0.105	0.291	159,719.303	0.333	102
AT1-AAs	LIGHT	0.128	-0.074	0.32	154,242.493	0.2	102
AT1-AAs	27-OHC	-0.107	-0.301	0.095	195,822.054	0.283	102
ACE2-AAs	IL-6	-0.02	-0.225	0.187	150,409.292	0.846	96
ACE2-AAs	IL-17	0.143	-0.066	0.34	122,417.027	0.166	95
ACE2-AAs	TNF-α	0.12	-0.089	0.318	129,784.962	0.245	96
ACE2-AAs	LIGHT	0.03	-0.178	0.234	143,071.915	0.774	96
ACE2-AAs	27-OHC	-0.017	-0.222	0.19	149,894.000	0.872	96
AT2-AAs	IL-6	0.079	-0.129	0.281	135,774.050	0.444	96
AT2-AAs	IL-17	0.04	-0.169	0.245	137,211.979	0.703	95
AT2-AAs	TNF-α	0.007	-0.2	0.213	146,430.369	0.947	96
AT2-AAs	LIGHT	0.003	-0.203	0.209	146,947.255	0.974	96
AT2-AAs	27-OHC	-0.03	-0.235	0.177	151,907.518	0.769	96
MasR-AAs	IL-6	0.075	-0.139	0.282	116,192.898	0.481	91
MasR-AAs	IL-17	0.146	-0.069	0.348	103,775.322	0.17	90
MasR-AAs	TNF-α	-0.052	-0.261	0.161	132,152.901	0.622	91
MasR-AAs	LIGHT	0.15	-0.064	0.35	106,789.879	0.157	91
MasR-AAs	27-OHC	-0.056	-0.265	0.157	132,641.575	0.597	91
IL-6	IL17	0.106	-0.097	0.301	153,463.274	0.29	101
IL-6	TNF-α	0.409	0.228	0.563	104,522.680	<0.001	102
IL-6	LIGHT	0.095	-0.107	0.289	160,078.979	0.343	102
IL-6	27-OHC	0.006	-0.195	0.206	175,844.332	0.955	102
IL-17	TNF-α	0.259	0.061	0.437	127,299.419	0.009	101
IL-17	LIGHT	0.52	0.356	0.653	82,396.736	<0.001	101
IL-17	27-OHC	0.026	-0.176	0.226	167,172.953	0.794	101
TNF-α	LIGHT	0.294	0.1	0.467	124,825.165	0.003	102
TNF-α	27-OHC	0.045	-0.157	0.242	168,969.471	0.656	102
LIGHT	27-OHC	0.12	-0.082	0.312	155,684.207	0.231	102

Spearman correlation coefficients with 95% confidence intervals, S statistics, and associated p-values for pairwise associations among RAS-related autoantibodies, inflammatory cytokines, and 27-hydroxycholesterol in the full control cohort. N indicates the number of valid pairwise observations. Statistically significant *p*-values are denoted in red.

Table S12. Pairwise Spearman correlation analysis among RAS-related autoantibodies, inflammatory cytokines, and 27-hydroxycholesterol in the full parkinsonian cohort.

Variable 1	Variable 2	Correlation Coefficient	95% CI Lower Limit	95% CI Upper Limit	S Statistic	p-value	N
AT1-AAs	IL-6	0.103	-0.086	0.285	233,332.315	0.271	116
AT1-AAs	IL-17	0.197	0.01	0.371	208,846.629	0.034	116
AT1-AAs	TNF-α	0.202	0.015	0.375	207,639.584	0.03	116
AT1-AAs	LIGHT	0.359	0.184	0.512	166,802.407	<0.001	116
AT1-AAs	27-OHC	0.347	0.17	0.502	169,904.827	<0.001	116
ACE2-AAs	IL-6	0.175	-0.013	0.351	214,583.626	0.06	116
ACE2-AAs	IL-17	0.068	-0.121	0.252	242,527.374	0.47	116
ACE2-AAs	TNF-α	0.08	-0.11	0.263	239,417.306	0.396	116
ACE2-AAs	LIGHT	-0.011	-0.198	0.177	262,952.508	0.908	116
ACE2-AAs	27-OHC	0.109	-0.08	0.291	231,776.000	0.244	116
AT2-AAs	IL-6	0.185	-0.003	0.361	206,483.839	0.047	115
AT2-AAs	IL-17	0.095	-0.096	0.278	229,504.447	0.315	115
AT2-AAs	TNF-α	0.065	-0.125	0.251	236,922.464	0.488	115
AT2-AAs	LIGHT	0.134	-0.055	0.315	219,393.938	0.152	115
AT2-AAs	27-OHC	0.141	-0.049	0.321	217,690.000	0.132	115
MasR-AAs	IL-6	0.011	-0.178	0.199	250,776.058	0.911	115
MasR-AAs	IL-17	0.138	-0.052	0.318	218,496.750	0.142	115
MasR-AAs	TNF-a	0.059	-0.13	0.245	238,395.079	0.528	115
MasR-AAs	LIGHT	0.117	-0.073	0.298	223,917.967	0.215	115
MasR-AAs	27-OHC	0.153	-0.037	0.331	214,802.255	0.104	115
IL-6	IL17	0.144	-0.045	0.323	222,616.277	0.122	116
IL-6	TNF-α	0.335	0.157	0.492	173,008.046	<0.001	116
IL-6	LIGHT	0.056	-0.133	0.241	245,600.874	0.552	116
IL-6	27-OHC	0.154	-0.034	0.332	219,970.563	0.098	116
IL-17	TNF-α	0.256	0.071	0.423	193,659.468	0.006	116
IL-17	LIGHT	0.509	0.356	0.636	127,686.712	<0.001	116
IL-17	27-OHC	-0.002	-0.19	0.185	260,743.881	0.98	116
TNF-α	LIGHT	0.18	-0.008	0.355	213,394.131	0.054	116
TNF-α	27-OHC	0.115	-0.074	0.296	230,168.116	0.218	116
LIGHT	27-OHC	0.152	-0.037	0.33	220,608.889	0.104	116

Spearman correlation coefficients with 95% confidence intervals, S statistics, and associated p-values for pairwise associations among RAS-related autoantibodies, inflammatory cytokines, and 27-hydroxycholesterol in the full parkinsonian cohort. N indicates the number of valid pairwise observations.Statistically significant *p*-values are denoted in red.

Table S13. Binary logistic regression analysis of the association between selected variables and Parkinson's disease status. Full model.

Variable	Category	Reference	Reg. Coeff.	Std. Error	LL (95% CI)	UL (95% CI)	z-value	p-value	Significance
Intercept			-4.245	1.415	-7.134	-1.56	-3	0.003	p<0.01 **
AT1-AAs			0.057	0.029	0.01	0.124	1.961	0.05	p<0.05 *
ACE2-AAs			0.007	0.008	-0.003	0.026	0.982	0.326	Not significant
MasR-AAs			0.009	0.005	-0.001	0.02	1.693	0.09	Not significant
Extraction-Age			0.05	0.019	0.013	0.089	2.574	0.01	p<0.05 *
SEX	Women	Men	0.084	0.347	-0.597	0.766	0.242	0.809	Not significant

Regression coefficients (Reg. Coeff.), standard errors (Std. Error), lower and upper limits of the 95% confidence interval (LL and UL, respectively), z-values, and p-values for each predictor included in the logistic regression model. The reference category for the variable SEX is "men." Statistical significance is indicated as follows: * ($p < 0.05$), ** ($p < 0.01$). AT1-AAs: Autoantibodies for AT1; ACE2-AAs: Autoantibodies for ACE2. MasR-AAs: Autoantibodies for MasR autoantibodies. Statistically significant *p*-values are denoted in red.

Table S14. Binary logistic regression analysis of the association between selected variables and Parkinson's disease status. Reduced model.

Variable	Category	Reference	Reg. Coeff.	Std. Error	LL (95% CI)	UL (95% CI)	z-value	p-value	Significance
Intercept			-4.387	1.408	-7.265	-1.72	-3.117	0.002	p<0.01 **
AT1-AAs			0.062	0.029	0.014	0.128	2.116	0.034	p<0.05 *
MasR-AAs			0.01	0.005	0	0.02	1.786	0.074	Not significant
Extraction-Age			0.053	0.019	0.017	0.092	2.791	0.005	p<0.01 **

Regression coefficients (Reg. Coeff.), standard errors (Std. Error), lower and upper limits of the 95% confidence interval (LL and UL, respectively), z-values, and p-values for each predictor included in the logistic regression model. Statistical significance is indicated as follows: * ($p < 0.05$), ** ($p < 0.01$). AT1-AAs: Autoantibodies for AT1; MasR-AAs: Autoantibodies for MasR; autoantibodies. Statistically significant *p*-values are denoted in red.
